# Porous Carbon Coated on Cadmium Sulfide-Decorated Zinc Oxide Nanorod Photocathodes for Photo-accelerated Zinc Ion Capacitors

**DOI:** 10.1021/acsami.2c20995

**Published:** 2023-01-27

**Authors:** Xiaopeng Liu, Holly Andersen, Yinan Lu, Bo Wen, Ivan P. Parkin, Michael De Volder, Buddha Deka Boruah

**Affiliations:** † Institute for Materials Discovery, University College London, London WC1E 7JE, UK; ‡ Department of Engineering, University of Cambridge, Cambridge CB3 0FS, UK; § Department of Chemistry, University College London, London WC1H 0AJ, UK

**Keywords:** porous carbon, heterostructure, light−matter interaction, photocathodes, zinc ion capacitors

## Abstract

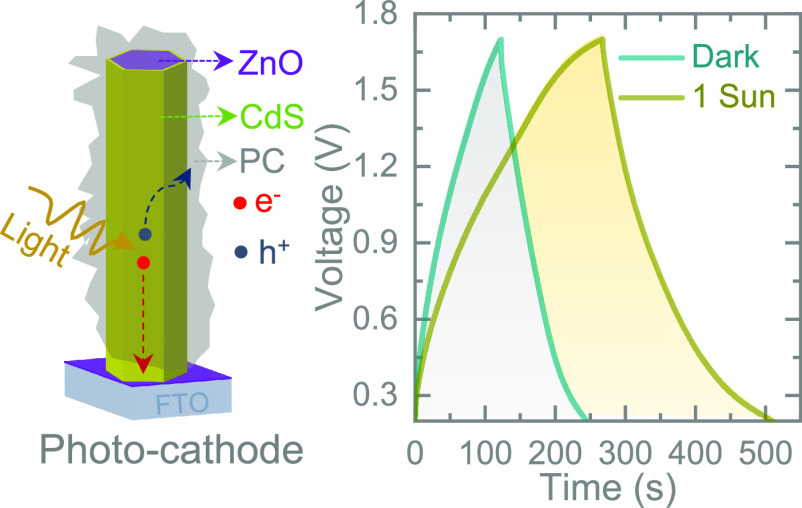

The development of devices with dual solar energy-harvesting and storage functionalities has recently gained significant traction for off-grid power supply. In their most compact embodiment, these devices rely on the same electrode to harvest and store energy; however, in this approach, the development of energy-efficient photoelectrodes with intrinsic characteristics of good optical and electrochemical activities remains challenging. Here, we propose photoelectrodes with a porous carbon coated on a zinc oxide–cadmium sulfide heterostructure as an energy-efficient photocathode for photo-accelerated zinc ion capacitors (Photo-ZICs). The Photo-ZICs harvest light energy and store charge simultaneously, resulting in efficient charge storage performance under illumination compared to dark conditions (∼99% capacity enhancement at 500 mA g^–1^ under illumination compared to dark conditions). The light absorption ability and charge separation efficiency achieved by the photocathodes meet the requirements for photo-ZIC applications. Moreover, Photo-ZICs display stable charge storage capacities over long-term cycling, that is, ∼1% capacity loss after 10,000 cycles.

## Introduction

Carbon emissions and their impact on climate change are major global concerns. Renewable energy sources such as solar energy are attractive alternatives to fossil fuels, but their intermittent availability makes their widespread use challenging. Therefore, the design of energy storage devices to balance energy supply and demand is of significant importance. The most common solution is the integration of a photovoltaic component with pumped hydro or electrochemical energy storage devices.^1−4^ For instance, Guo et al. presented a solar rechargeable battery system based on a dye-sensitized solar cell and a lithium-ion battery,^5^ Li et al. integrated a photovoltaic flow battery with a perovskite/silicon tandem solar cell,^6^ and Chen et al. integrated a dye-sensitized solar cell and electrochemical capacitor system.^7^ However, these kinds of systems tend to suffer from issues such as ohmic transport losses, energy mismatch between energy harvester and storage components, and high fabrication costs. One approach to address some of these obstacles is the development of photocathodes that combine solar energy-harvesting and storage. These photo-assisted energy storage systems coupled photovoltaic and energy storage capabilities simultaneously, reducing the fabrication cost. Moreover, this “2 in 1” concept increases the gravimetric energy storage performance and avoids the loss of energy transfer between discrete modules. Until now, photo-induced promotion has been widely used in various electrochemical energy storage devices, including Li–O_2_ batteries,^8^ Li–S batteries,^9^ Li-ion batteries,^10^ and Li–CO_2_ battery.^11^ Although Li-based materials dominate the market of electric energy storage devices (batteries and electrochemical capacitors), the high cost of lithium and the safety concerns relating to the metal may prohibit its use for certain applications. Therefore, where cost efficiency is a priority, alternative energy storage systems need to be explored to fulfill these requirements.

Given the evident advantages of low price, excellent safety, high specific capacity, and long-term stability, aqueous zinc ion capacitors (ZICs) have recently been proposed as one of the most promising energy storage technologies. Earlier, we explored V_2_O_5_^12^ and g-C_3_N_4_^13^ photoelectrodes for photo-accelerated ZICs (Photo-ZICs) where photocathodes were fabricated by physical mixing of active materials with charge transfer materials, a conductive additive, and a binder. However, the random mixing of functional materials with charge transfer materials may limit the photo-charge separation and transportation characteristics.^14,15^ Further, inefficient optical activities of bifunctional electrode materials may suffer from limited solar energy conversion efficiency. This work presents a porous carbon (PC) coated on cadmium sulfide-decorated zinc oxide nanorod array (PC/CdS@ZnO NRs; Figure S1) photocathode for a Photo-ZIC. Here, the combination of CdS and ZnO promote photoconversion efficiency by heterojunction formation. PC is used to further separate photoinduced electron–hole pairs and to facilitate charge transfer and storage.

## Results and Discussion

**Figure 1:**
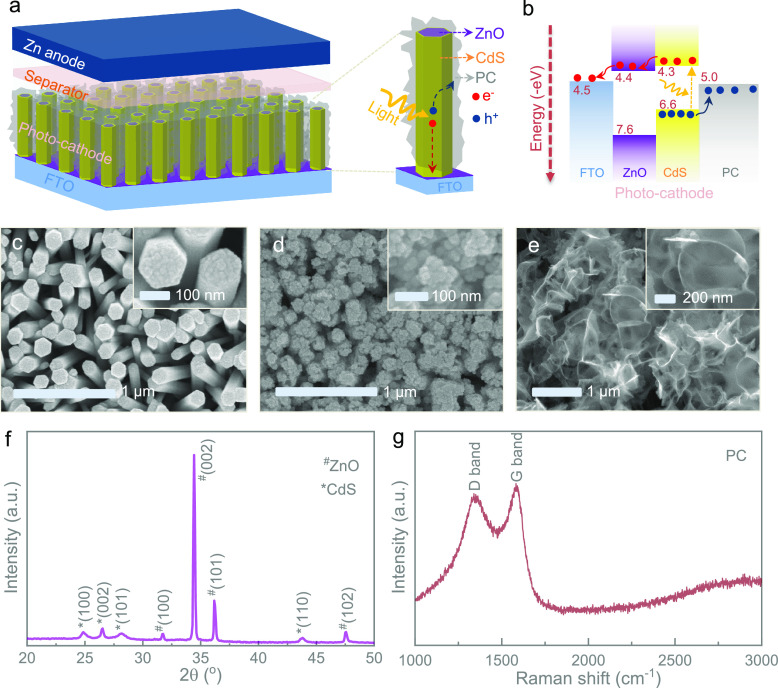
(a) Schematic representation of the proposed Photo-ZIC using the PC/CdS@ZnO NRs photocathode and Zn anode. The enlarged image shows the possible photo-charge generation and transportation. (b) Energy band diagram of the photocathode illustrating the formation of a heterojunction between ZnO and CdS with possible photo-charge separation and separation due to the generation of built-in electric fields at the interfaces. (c, d) SEM images of as-grown ZnO NRs and CdS-decorated ZnO NRs. (e) SEM image of the as-synthesized PC. (f) XRD pattern of CdS@ZnO NRs. (g) Raman spectrum of PC.

Figure 1a schematically depicts the configuration of the proposed Photo-ZIC. Under illumination, the photogenerated electrons transport from CdS to FTO through ZnO due to an energetically favorable pathway and finally to the Zn anode through the external circuit to deposit Zn^2+^ ions from the electrolyte. On the other hand, the photogenerated holes transport to the PC to form electrical double-layer capacitance to allow photo-charging. The detailed illustration in Figure 1b is a representation of the heterojunction formed between ZnO and CdS. Because of the difference in band positions, it is favorable for the electrons in CdS to drift to ZnO, thus generating a built-in electric field whose direction is from CdS to ZnO. When the device is illuminated, this heterojunction drives the transfer of photogenerated electrons from the conduction band of CdS to the conduction band of ZnO, reducing the recombination of photogenerated electron–hole pairs within the CdS semiconductor. As a result, the photoconversion efficiency of the system is improved. Moreover, the presence of the PC coating provides a transfer path for charge carriers, which facilitates charge separation and improves the system’s photoactivity and simultaneously storage energy via an electrical double-layer capacitor.

SEM analysis was carried out on the synthesized samples (Figure 1c–e). It can be seen that ZnO samples show an ordered rod-like hexagonal morphology with approximately 140–160 nm in diameter (Figure 1c). After being coated with CdS, the particles are agglomerated on the surface of ZnO NRs (Figure 1d). Figure 1e shows the SEM image of an as-synthesized PC. XRD confirms the crystal structure of the samples in Figure 1f. The diffraction peaks of 31.8, 34.5, 36.3, and 47.6° can be indexed to the (100), (002), (101), and (102) crystal planes of hexagonal wurtzite ZnO (JCPDS no. 36-1451), respectively (Figure S2a,b shows the XRD patterns of pristine ZnO and the PC/CdS@ZnO NRs photocathode).^16^ Meanwhile, the characteristic peaks associated with the (100), (002), (101), and (110) planes of hexagonal CdS (JCPDS no. 41-1049) indicate that CdS has been successfully composited with ZnO.^17^ Raman spectra in Figure 1g show the wide D and G bands around 1350 and 1580 cm^-1^, which are characteristic peaks of amorphous carbon with a D/G intensity ratio of 0.94. The appearance of the peaks could be attributed to the disordered sp^3^-hybridized carbon and ordered graphitic sp^2^-hybridized carbon. Moreover, Figure S2c shows the cross-sectional SEM image of CdS@ZnO NRs, whereas the top-view SEM image of drop-cast PC on CdS@ZnO NRs is shown in Figure S2d. Moreover, the UV–vis spectrum of the photocathode (Figure S3) demonstrates the band-gap energy of CdS at approximately ∼2.3 eV.

**Figure 2:**
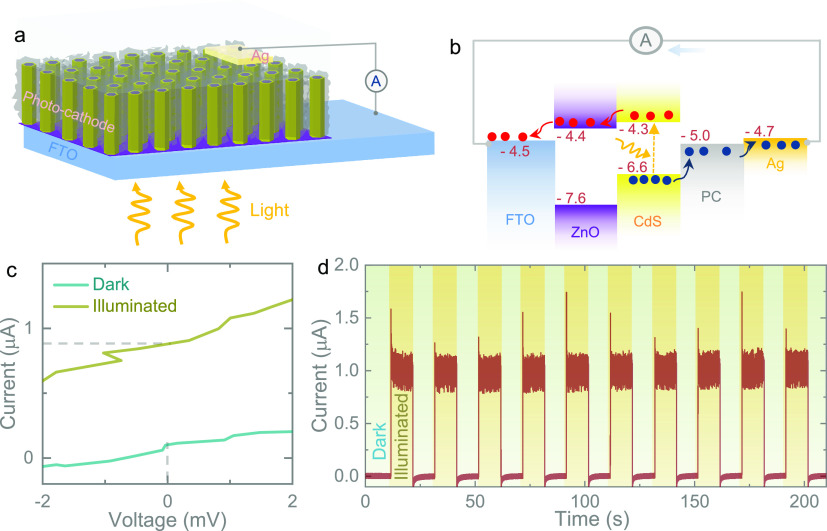
(a, b) Schematic illustration of a silver (Ag)/PC/CdS@ZnO NRs/FTO-based layer-by-layer photodetector and its energy band diagram. (c) Current–voltage curves of the photodetector in dark and illuminated (λ ≈ 455 nm) conditions. (d) Current (photo-current–dark current)–time plot of the photodetector in the absence of an applied bias voltage (0 V) under periodic illumination (λ ≈ 455 nm).

In order to reveal the optoelectronic characteristics of the photocathode, a self-powered photodetector device formed with a ZnO@CdS NR/PC/Ag heterojunction on a transparent FTO substrate was fabricated. A scheme of this device is shown in Figure 2a, while Figure 2b illustrates the energy band diagram of the photodetector. Detailed fabrication processes can be found in the Experimental Section (Supporting Information). Figure 2c shows the *I*–*V* characteristics of the fabricated photodetector in dark and illuminated (λ ≈ 455 nm) conditions. This signifies that the photodetector shows a better current under illumination, which affirms the photosensitivity of the photocathode. Even at a 0 V bias voltage, the photodetector shows a current response confirming that the heterojunctions of the photocathodes enable the separation of photogenerated electrons and holes to generate a photo-current (as illustrated in Figure 2a) even without an external driving force. Figure 2d (current–time plot under periodic illumination) further confirms the photo-current generation under illumination without an external bias voltage. The current signal immediately rises and equilibrates under illumination and drops once the light is turned off. The observed drop to negative currents may be attributed to the pyro-phototronic effect of ZnO NRs.^18^

**Figure 3:**
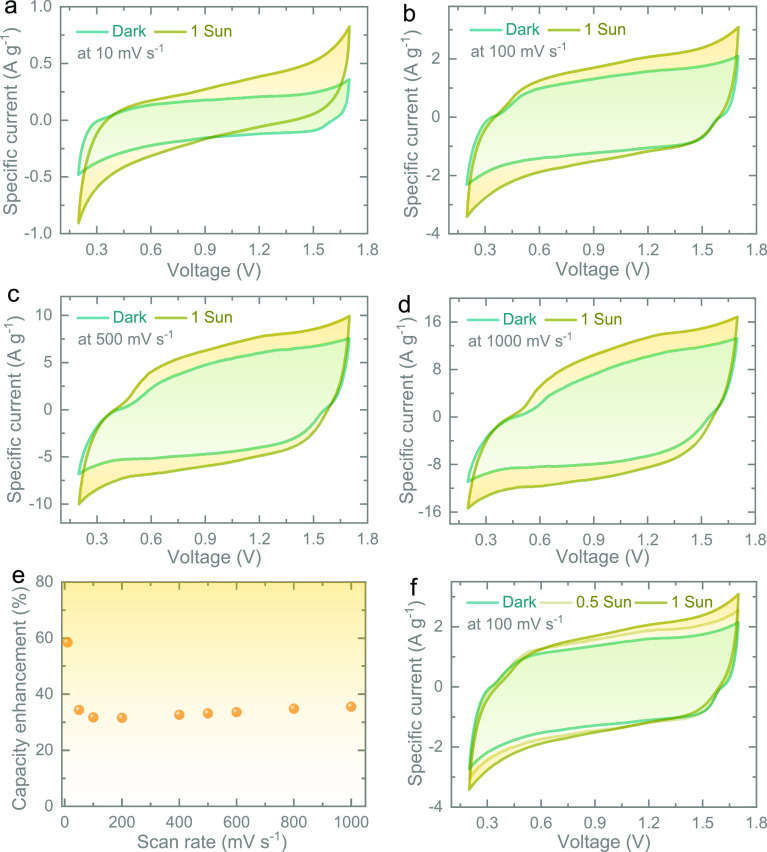
Comparative CV curves at scan rates of (a) 10 mV s^–1^, (b) 100 mV s^–1^, (c) 500 mV s^–1^, and (d) 1000 mV s^–1^ in the dark and 1 sun illumination. (e) Capacity enhancement with a scan rate plot. (f) Comparative CVs at 100 mV s^–1^ in the dark and 0.5 sun and 1 sun illuminations.

Next, the electrochemical performance of the photocathodes was tested against a zinc anode using 2045-type optical coin cells in dark and illuminated (1 sun) conditions (see the Experimental Section). Cyclic voltammetry (CV) measurements were carried out over a voltage window between 0.2 and 1.7 V at various scan rates of 10–1000 mV s^–1^ (Figure 3a–d and Figure S4). It is shown that light illumination effectively improves the charge storage performance of the Photo-ZICs. Figure 3e demonstrates the capacity enhancement at different scan rates in the dark and 1 sun illumination. The larger capacities of the photocapacitors under light than of dark conditions could be attributed to the photo-charging effect where photo-charge carriers participate in the charge storage process under illumination. The highest capacity enhancement of ∼59% is achieved at a slow scan rate (10 mV s^–1^), while improvements of approximately 30–35% are achieved at faster scan rates. To illustrate the role of light intensity, comparative CVs under different illumination conditions are provided in Figure 3f, which shows a decrease in capacity as the light intensity decreases. This is because, with an increasing light intensity, the rate of photo-charge generation increases, resulting in more photo-charges available to participate in the charge storage process.

**Figure 4:**
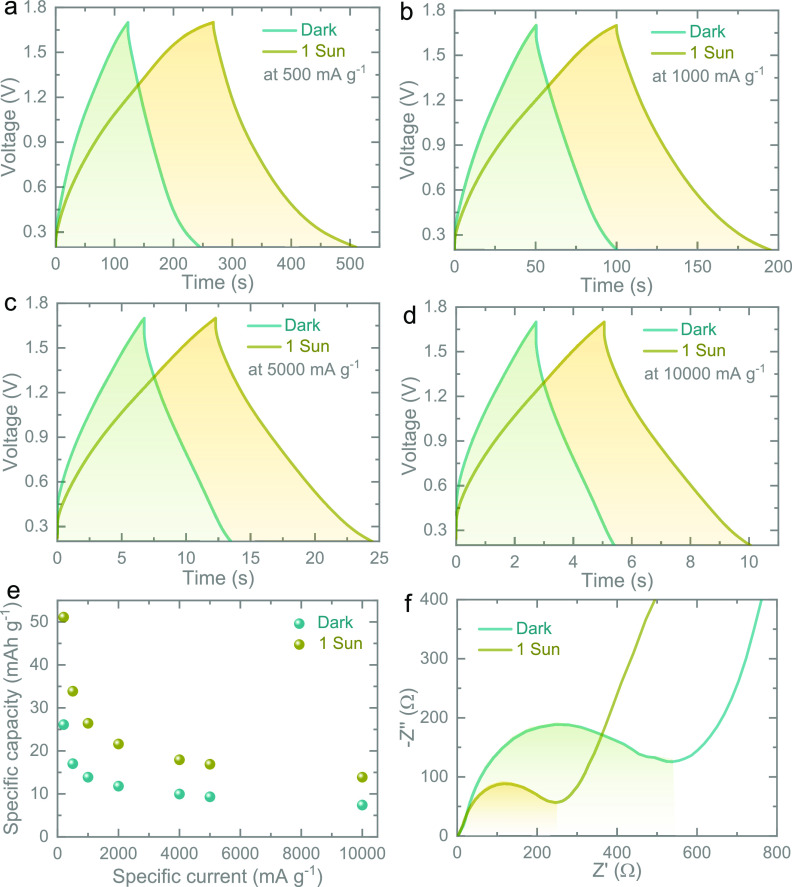
Comparative CD curves at specific currents of (a) 500 mA g^–1^, (b) 1000 mA g^–1^, (c) 5000 mA g^–1^, and (d) 10,000 mA g^–1^ in the dark and 1 sun illumination. (e) Capacity concerning the specific current plot in the dark and 1 sun illuminated conditions. (f) Nyquist plots in the dark and 1 sun illumination.

The electrochemical behaviors of the photocathodes were further assessed using galvanostatic charge/discharge tests (CD) in dark and illuminated conditions (Figure 4a–d and Figure S5). The corresponding capacity enhancements under different scan rates are summarized in Figure 4e. For instance, capacity improvements due to illumination of 99% at 500 mA g^–1^ and 86% at 10000 mA g^–1^ are observed. The difference in capacity improvements under a series of specific currents could be ascribed to the light interaction and photo-generated charge participation in the charge storage process. Under illumination, the photoexcited electrons transport from CdS to FTO through ZnO, whereas the remaining photogenerated holes move to PC due to favorable energy pathways to contribute to the charge storage process (Figure 1b). The electrochemical impedance spectra (EIS) measured in dark and illuminated conditions are shown in Figure 4f. The Nyquist plots consist of two parts: (i) The straight line slope at low frequency is inversely related to the ionic resistance due to the diffusion process. (ii) The semicircle diameter at a high frequency positively corresponds to charge transfer resistance (∼534 and ∼245 Ω in dark and illuminated conditions). It can be seen that the photocathode offers a lower charge transfer resistance when exposed to light as the semicircle diameter in the illumination condition is smaller than that in the dark state. This is probably due to additional charge carriers being generated under the light, which causes a reduction in impedance. However, a negligible change in equivalent series resistance increases is observed in dark (∼2.8 Ω) and illuminated (∼2.1 Ω) conditions. Figure S6 shows the Bode plots in the dark and 1 sun illumination, demonstrating peak frequency values of ∼240 and ∼435 Hz. Shifting the peak frequency value to a higher frequency under illumination compared to in the dark indicates improvement in the diffusive resistance.

**Figure 5:**
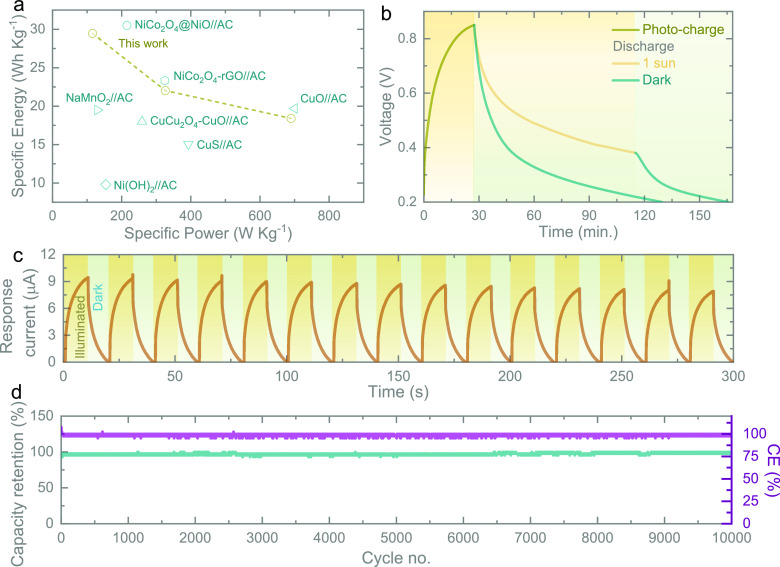
(a) The Ragone plot demonstrates the comparison of the specific energy and power of our Photo-ZIC with those reported for asymmetric supercapacitors in the literature: NiCo_2_O_4_-rGO∥activated carbon (AC),^19^ NiCo_2_O_4_@NiO∥AC,^20^ CuO∥AC,^21^ NaMnO_2_∥AC,^22^ CuCo_2_O_4_-CuO∥AC,^23^ CuS∥AC,^24^ and Ni(OH)_2_∥AC.^25^ (b) Photo-charge and discharge at 0.02 mA cm^–2^ in dark and illuminated conditions. (c) Response current (photo-current–dark current) plot of the Photo-ZIC under periodic illuminated and dark cycles at OCP (∼1.05 V). (d) Long-term cycling test at 5000 mA g^–1^ of the Photo-ZIC in the dark.

Figure 5a shows a Ragone plot of the specific energy and specific power of asymmetric supercapacitors. It shows that, in addition to its photo-enhanced properties, our capacitor also achieves energy storage performance comparable to advanced asymmetric supercapacitors reported in the literature. The light-charging and discharge capability of our Photo-ZIC is studied in Figure 5b. First, we discharged the cell in dark conditions and then under illumination. The latter results in a slower voltage drop because the cell is light-charged at the same time as being discharged. After the light source is removed, the voltage output drops to 0 V rapidly in a similar fashion to dark discharge measurements. Further, the self-discharges of the Photo-ZICs in the dark and 1 sun illumination (Figure S7) confirm a slow self-discharge rate under illumination due to the simultaneous photo-charging effect compared to that of the dark. Further, the response current plot (Figure 5c) of the Photo-ZIC at OCP (∼1.05 V) shows the increase in the response under illumination because of the transportation of photo-generated electrons through the external circuit. To illustrate the cycling properties of the Photo-ZIC, charge/discharge cycling tests are conducted at 5000 mA g^–1^ in dark conditions (Figure 5d). The capacity of the photo-ZIC remains stable over cycling with a capacity retention of ∼99% and high Coulombic efficiency (∼100%) after 10,000 cycles, which shows good cycling stability under dark conditions.

## Conclusions

In summary, we propose a strategy to design high-performance Photo-ZICs where energy is stored in the double layer of a PC layer and photo-charges are generated and separated in CdS/ZnO photocathodes. This heterostructure allows for light-harvesting and charge transfer of holes to the PC layer, which allows for charging the capacitor. Meanwhile, the active PC material allows for a reversible and high charge storage capacity with superior long-term stability of only an ∼1% capacity loss after 10,000 cycles. Therefore, we believe that this photocathode design may provide a promising route for the design of energy-efficient photocathodes for future photo-accelerated energy storage applications.

## Experimental Section

### Chemicals

Zinc acetate dihydrate (Zn(CH_3_COO)_2_·2H_2_O), *N*,*N*-dimethylmethanamide (DMF), zinc nitrate hexahydrate (Zn(NO_3_)_2_·6H_2_O), hexamethylenetetramine ((CH_2_)_6_N_4_), cadmium nitrate tetrahydrate (Cd(NO_3_)_2_·4H_2_O), thiourea (CH_4_N_2_S), porous carbon, SuperP carbon black, polyvinylidene fluoride (PVDF), and *N*-methyl-2-pyrrolidone (NMP) were purchased and utilized as received.

### Synthesis of PC

PC was obtained by directly annealing 5 g of sodium citrate at 700 °C for 1 h in the presence of argon gas (400 sccm). The received black product was washed using hot water (100 °C) to remove the salt followed by drying at 60 °C in a vacuum oven.

### Synthesis of ZnO NPs

An amount of 100 mg of zinc acetate dihydrate was dispersed into 100 mL of DMF using sonication and vortex mixing processes. The clear solution was then sustained at 105 °C for 5 h. The FTO glass substrate (received from Sigma-Aldrich; surface resistivity ≈ 7 Ω sq.^–1^) was cleaned with isopropyl alcohol, ethanol, and deionized (DI) water followed by UV ozone treatment for 20 min. The ZnO NPs seed layer was prepared on an FTO glass substrate via spin-coating (1000 rpm, 1 min) and dried at 150 °C for 5 min. The process was repeated five times to obtain a uniform ZnO NPs seed layer, which was then thermally treated at 250 °C for 1 h in air.

### Synthesis of ZnO NRs

The ZnO NPs seed layer-coated FTO substrate was dipped into a 200 mL solution of equimolar 25 mM zinc nitrate hexahydrate and hexamethylenetetramine with the seed layer-coated side facing down. The mixture was heated at 95 °C for 15 h and then was cleaned with DI water and ethanol followed by drying at 120 °C for 1 h in air.

### Synthesis of CdS@ZnO NRs

The prepared ZnO NRs grown on FTO was immersed in a 240 mL solution of 1 mmol of cadmium nitrate and 3 mmol of thiourea with the coated side facing down. The mixture was heated at 180 °C for 5 h and then was cleaned with DI water and ethanol followed by drying at 120 °C for 1 h in air.

### Preparation of the PC/CdS@ZnO NRs Photocathode

To prepare the photocathode, 40 mg of PC was mixed well with 10 wt % SuperP and 10 wt % PVDF binder in 2 mL f *N*-methyl-2-pyrrolidone by sonication and vortex mixing processes for 2 h. The solution was then cast on the various substrates, including CdS@ZnO NRs/FTO or ZnO NRs/FTO or clean FTO, followed by drying at 80 °C for 12 h.

### Characterizations

The crystalline samples were examined using XRD and Raman analysis. SEM investigated the surface morphology of the samples. Further, the optical properties of the samples were analyzed using UV–vis–NIR spectroscopy.

### Design of the Photo-ZICs

The 2045-type optical coin cells were assembled using an as-prepared photocathode (18 mm diameter) and a Zn metal anode (14 mm diameter) on a Whatman glass microfiber filter paper separator (19 mm diameter) with the addition of 150 μL of 3 M Zn(CF_3_SO_3_)_2_ aqueous electrolyte. However, an 8 mm-diameter hole was designed as an optical window in the center of the cathode case. The photocathode was fixed using carbon fibers, and melted parafilm allowed light illumination before assembling the optical cell.

### Electrochemical Tests

CV and CD measurements of the Photo-ZICs were conducted at different scan rates ranging from 10 to 1000 mV s^–1^ (voltage window of 0.2–1.7 V) and specific currents ranging from 500 to 10,000 mA g^–1^ (voltage window of 0.2–1.7 V) using a galvanostatic battery tester (Biologic VMP-3) in dark and illuminated conditions. EIS tests of the Photo-ZIC were tested in the frequency range from 10 mHz to 100 kHz at a voltage amplitude of 10 mV in dark and illuminated conditions. Further, a Neware battery testing system was used to perform the cycling GCD measurements at different specific current rates under dark conditions.

### Fabrication of Photodetector and Electrical Measurements

A layer-by-layer Ag/PC/CdS@ZnO NRs/FTO photodetector was fabricated by drop-casting pristine PC on the CdS@ZnO NRs/FTO substrate followed by drying at 80 °C for 10 h in air. A Ag contact on top of PC was used to probe the photodetector across FTO, and the electrical photoresponses (current–voltage in the sweeping voltage from −2 to +4 mV in dark and illuminated conditions and current–time in the absence of an external bias voltage under periodic illumination) were recorded using a source measuring unit.

## Supporting Information Available

Experimental steps involved with the photocathode, XRD of ZnO NRs and PC/CdS@ZnO NRs, SEM images, UV–vis spectrum of the photocathode, CV curves, CD curves, Bode plots, and self-discharge curves (PDF)
