# Supplementary material for: Porous Carbon Coated on Cadmium Sulfide-Decorated Zinc Oxide Nanorod Photocathodes for Photo-accelerated Zinc Ion Capacitors
Source: ACS Appl Mater Interfaces. 2023 Jan 27;15(5):6963–9. doi: 10.1021/acsami.2c20995 (PMC9923686; doi:10.1021/acsami.2c20995)
Supplement: Supplementary file 1 — am2c20995_si_001.pdf [file am2c20995_si_001.pdf]

Supporting Information

For

Porous Carbon-coated on Cadmium Sulfide Decorated Zinc Oxide Nanorods Photo-cathodes for Photo-Accelerated Zinc Ion Capacitors

Xiaopeng Liu,[1] Holly Andersen,[1] Yinan Lu,[1] Bo Wen,[2,] Ivan P. Parkin,*[,3] Michael De Volder,*[,2] Buddha Deka Boruah*[,1]

[1]Institute for Materials Discovery, University College London, London WC1E 7JE, UK

[2]Department of Engineering, University of Cambridge, Cambridge CB3 0FS, UK

[3]Department of Chemistry, University College London, London WC1H 0AJ, UK

Corresponding Authors:

Prof. Ivan P. Parkin, E-mail: i.p.parkin@ucl.ac.uk

Prof. Michael De Volder, E-mail: mfld2@cam.ac.uk

Dr. Buddha Deka Boruah, E-mail: b.boruah@ucl.ac.uk

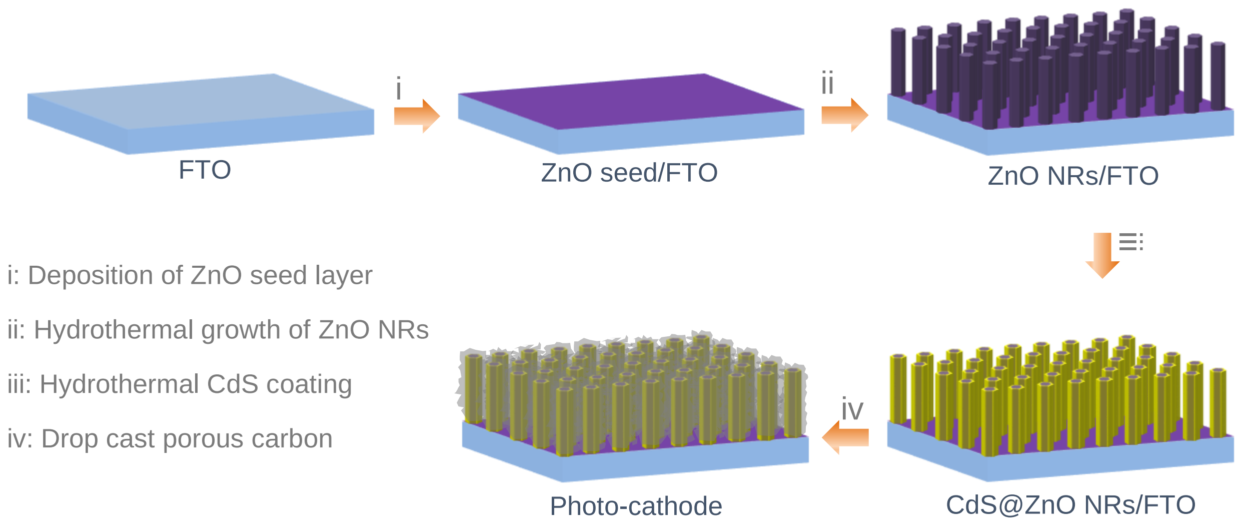

Figure S1. *Schematics show the experimental steps involved with the photo-cathode.*

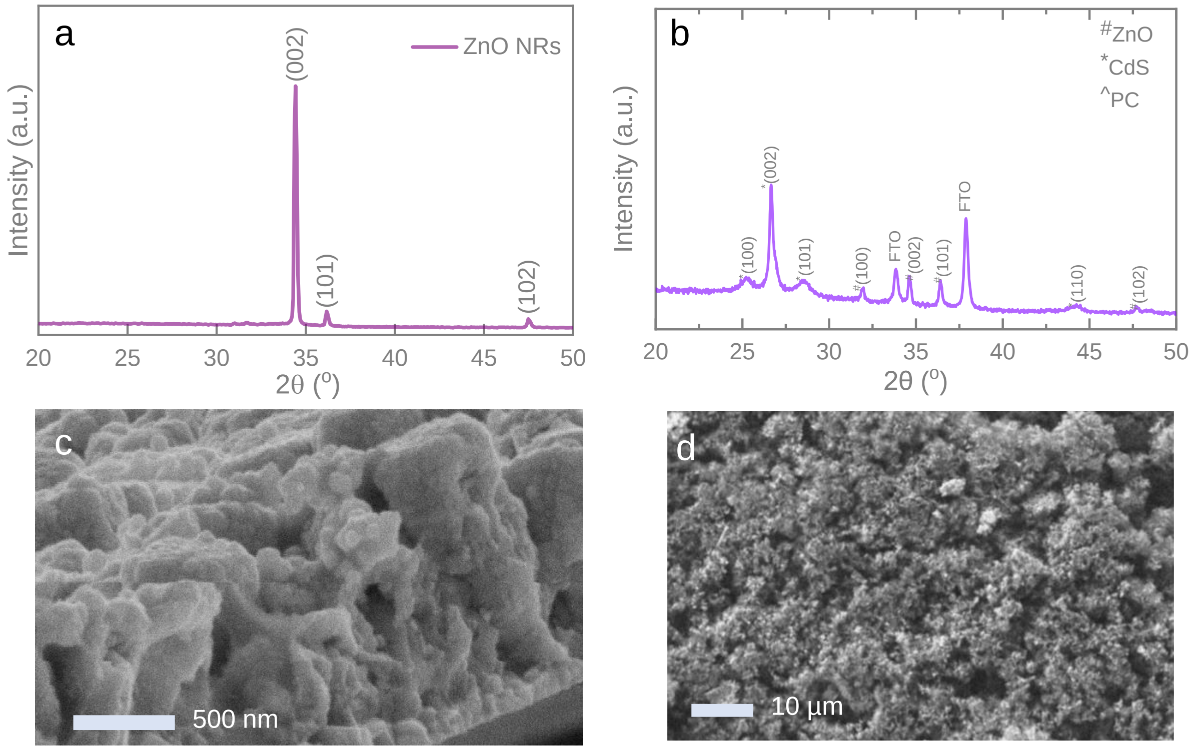

Figure S2. *(a, b) XRD pattern of ZnO NRs and PC/CdS@ZnO NRs. (c) Cross-section SEM image of CdS@ZnO NRs.(d) Top SEM image of PC/CdS@ZnO NRs.*

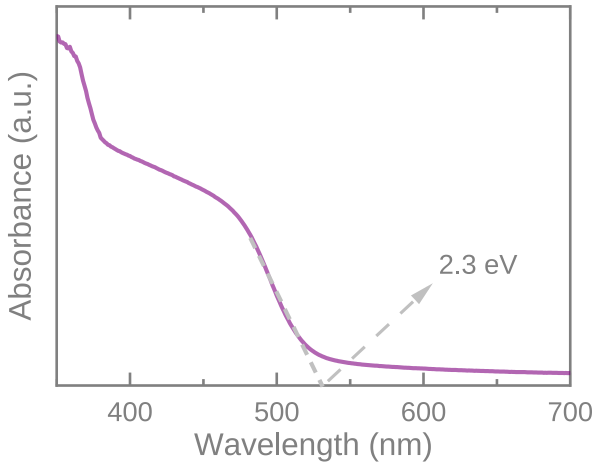

Figure S3. *UV-VIS spectrum of the photo-cathode.*

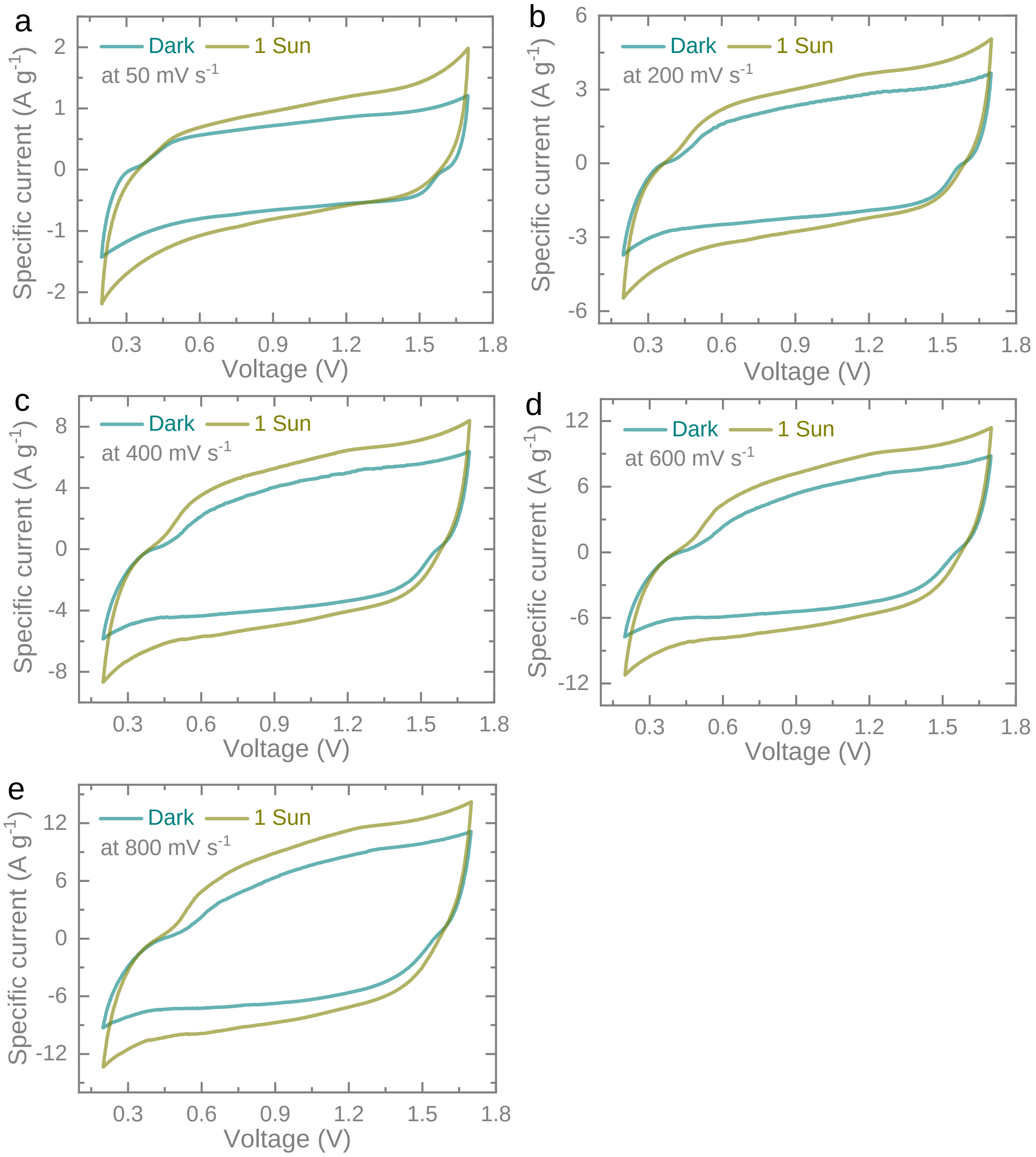

Figure S4. *Comparative CVs at different scan rates of (a) 50 mV s^{-1}, (b) 200 mV s^{-1}, (c) 400 mV s^{-1}, (d) 600 mV s^{-1} and (e) 800 mV s^{-1} in dark and 1 sun illuminated conditions.*

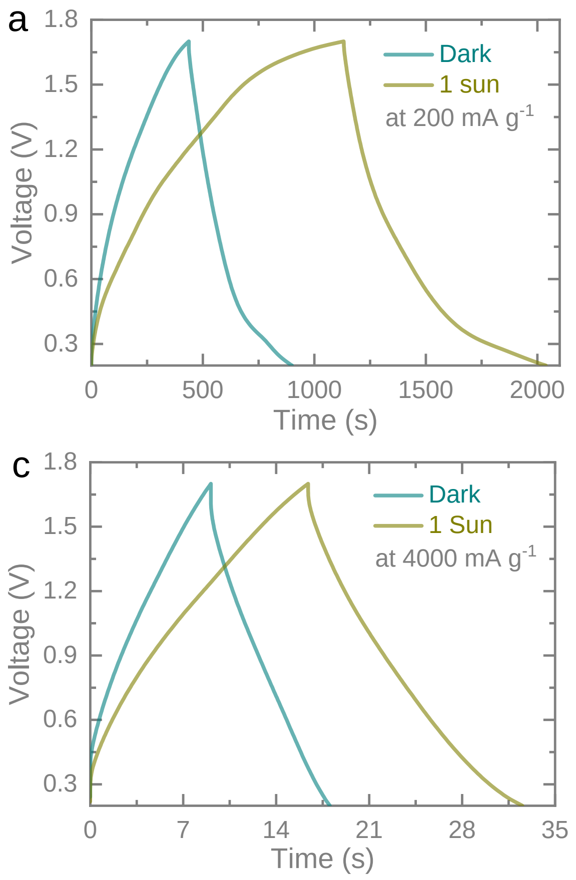

Figure S5. *CD curves at specific currents of (a) 200 mA g^{-1}, (b) 2000 mA g^{-1}, and (c) 4000 mA g^{-1} in dark and 1 sun illumination.*

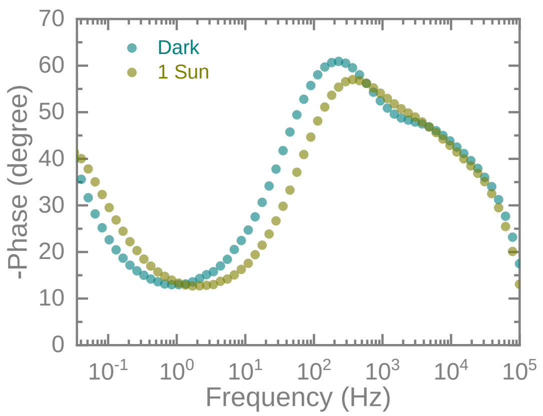

Figure S6. *Bode plots in the dark and 1 sun illumination.*

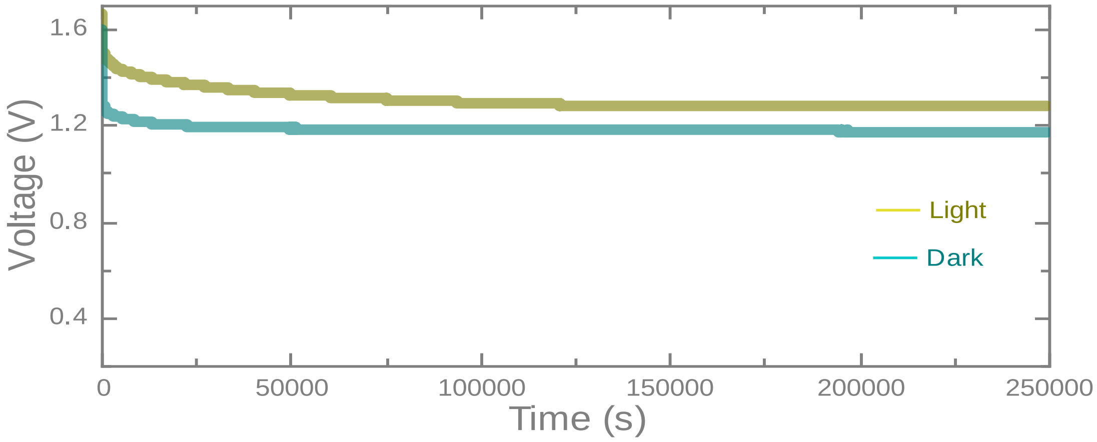

Figure S7. *Self-discharge behaviour in (a) dark and (b) illuminated conditions.*
